# Palliative Care Awareness among Indian Undergraduate Health Care Students: A Needs-Assessment Study to Determine Incorporation of Palliative Care Education in Undergraduate Medical, Nursing and Allied Health Education

**DOI:** 10.4103/0973-1075.73645

**Published:** 2010

**Authors:** Sakshi Sadhu, Naveen Sulakshan Salins, Asha Kamath

**Affiliations:** KMC Manipal, Manipal University, Manipal, India; 1 Palliative Medicine Consultant, Shiridi Saibaba Cancer Hospital and Research Center, KMC Manipal, Manipal, India; 2 Department of Community Medicine, KMC Manipal, Manipal University, Manipal, India

**Keywords:** Awareness, Education, Palliative care, Undergraduate

## Abstract

**Purpose:**

Quality assurance data worldwide suggests that the current healthcare system is providing inadequate care for the dying. Current health care education focuses entirely on cure and care is almost compromised or nonexistent in end-of-life settings. The purpose of this study was to determine palliative care awareness among Indian undergraduate health care students and assess the need for incorporating palliative medicine education into undergraduate health education.

**Materials and Methods:**

A non-randomized population based study was conducted using 39-point questionnaire. Undergraduate medical, nursing and allied health students of Manipal University were the target population.

**Results:**

326 students participated in the study. 61.7% of students feel that resuscitation is appropriate in advanced metastatic cancer. 67.5% feel that all dying patients need palliative care and most of the students think that palliative care is equivalent to pain medicine, geriatric medicine and rehabilitation medicine. 89% of students think that Morphine causes addiction in palliative care setting. 60.7% of students feel that prognosis should only be communicated to the family.

**Conclusion:**

The outcomes of the study showed that the basic knowledge of palliative care among students was inadequate, and students are unprepared and uncertain in their approach of delivering end-of-life care.

## INTRODUCTION

Majority of deaths in urban India occur in healthcare institutions and doctors either specialists or general practitioners will care for most of these patients. Despite a significant population of dying patients with palliative care needs, death and dying has been inadequately examined and addressed in health care training. Palliative care training is almost nonexistent in most of health care training program curricula. Studies have shown that doctors and other health care professionals lack knowledge and confidence in their ability to care for the dying patients, are not rewarded for exhibiting concern over psychosocial issues in end-of-life care, and are unwilling to administer adequate dosages of analgesics or sedatives to dying patients or to withhold or withdraw life support. An effective palliative care service delivery requires an informed health sector, with health care providers in all areas, aware and committed to the benefits that palliative care offers to people who are dying. Health professionals function largely within a culture that focuses on cure, and many avoid the patient who is dying. It is therefore vital that all health care practitioners who have contact with people who are dying, including medical, allied health science and nursing are aware and apply the best principles of palliative care. A needs assessment evaluates the educational need for a curriculum and possible content and instructional strategies. It also provides information so that appropriate and realistic implementation and evaluation plans can be developed.[1] This study helps to understand adequacies in various aspects of palliative care knowledge among undergraduate health care students and need to fill these gaps in knowledge by incorporating palliative care education in undergraduate medical, nursing and allied health education.

### Objective

To assess palliative care knowledge among undergraduate medical, nursing and allied health science students of Manipal University and evaluate need for incorporation of palliative care education in undergraduate medical, nursing and allied health education.

## MATERIALS AND METHODS

### Study design

Nonrandomized population based study conducted between June and August 2010 at Manipal University, Manipal, India. Target population for the study included undergraduate medical students (third and fourth year), undergraduate nursing students and undergraduate allied health students (physiotherapy and occupational therapy students). Kasturba Hospital Manipal ethics committee approval was obtained prior to the study. A 39-point questionnaire was prepared with 10 major subdivisions with three-four stems under each subdivision. This questionnaire was internally validated. A pretest informed consent was taken from every student participated in this study. 326 students participated in this study. Among the 326 students participated in the study, there were 18 undergraduate nursing students, 48 allied health students that included undergraduate students from both physiotherapy and occupation therapy streams and 260 medical students.

### Statistics

Data was entered in Microsoft excel and analyzed using SPSS ver 15(SPSS South Asia, Bangalore). Percentages were used to summarize the data. Chi square test was used to study the difference in proportions responded from each category of students. A P value of less than 0.05 was regarded as statistically significant.

## RESULTS

A total of 38.3% of students have answered correctly regarding appropriateness of resuscitation in advanced metastatic malignancy and majority of medical students (71.5%) students think that outcomes of resuscitation in advanced metastatic malignancy are not good. 63.5% of total students think that family should be involved in making decisions about resuscitation and this was higher among nursing students (88.9%) and the differences were statistically significant. More than half of students perceive that Indian legal system has laid down clear guidelines about end of life and resuscitation [Table 1].

**Table 1:** Resuscitation in cancer patients

Question – Appropriate response	Nursing *N*=18	Allied health *N*=48	Medical *N*=260	*P* value	Total *N*=326
Resuscitation is always appropriate in advanced metastatic cancer - No	7(38.9)	23(47.9)	95(36.5)	0.329	125(38.3)
Outcomes of resuscitation in advanced metastatic cancer are always good - No	9(50)	31(64.6)	186(71.5)	0.118	226(69.3)
Patients and relatives should always be involved in decision making of “not for resuscitation” - Yes	16(88.9)	33(68.8)	158(60.8)	0.040	207(63.5)
Indian legal system at present has laid down clear guidelines about end of life and not for resuscitation -No	4(22.2)	21(43.8)	110(42.3)	0.232	135(41.4)

Figures in parenthesis are in percentage

Two thirds of the students think that the philosophy of palliative care affirms life. 94.4% of nursing students feel that philosophy of palliative care recognizes dying as a normal process and the differences when compared were statistically significant. Less than one third of students understand that the philosophy of palliative care considers hastening of death and more than half of total students think that palliative care philosophy considers prolonging life [Table 2].

**Table 2:** Philosophy of palliative care

Question – Appropriate response	Nursing *N*=18	Allied health *N*=48	Medical *N*=260	*P* value	Total *N*=326
Affirms life –Yes	16(88.9)	34(70.8)	163(62.7)	0.054	213(65.3)
Recognizes dying as a normal process –Yes	17(94.4)	38(79.2)	182(70)	0.044	237(72.7)
Hastens death – No	11(61.1)	26(54.2)	194(74.6)	0.011	231(70.9)
Prolongs life – No	5(27.8)	19(39.6)	123(47.3)	0.194	147(45.1)

Figures in parenthesis are in percentage

Significant number of the students feels that all the dying patients need palliative care. Majority of students (76.7%) felt that palliative care is needed for uncontrolled pain in metastatic cancer. 56.4% of students felt that palliative care is useful in end stage heart failure. 46.9% of total students think that the palliative care is needed in acute postoperative pain but when individual disciplines were compared nursing students seem to have greater knowledge differentiating acute pain medicine and palliative medicine [Table 3].

**Table 3:** Palliative care is needed for

Question – Appropriate response	Nursing *N*=18	Allied health *N*=48	Medical *N*=260	*P* value	Total *N*=326
All dying patients - No	9(50)	17(35.4)	80(30.8)	0.217	106(32.5)
Metastatic cancer with uncontrolled pain -Yes	14(77.8)	33(68.8)	203(78.1)	0.371	250(76.7)
End stage heart failure - Yes	11(61.1)	30(62.5)	143(55)	0.578	184(56.4)
Three days after gall bladder surgery with pain - No	15(83.3)	19(39.6)	139(53.5)	0.006	173(53.1)

Figures in parenthesis are in percentage

Majority of students participated understand that palliative medicine is same as pain medicine and this knowledge was particularly poor among medical students. More than two thirds of students were unable to differentiate rehabilitation medicine, geriatric medicine from palliative care. However bulk of students relate active care of the dying with palliative care [Table 4].

**Table 4:** Palliative care is

Question – Appropriate response	Nursing *N*=18	Allied health *N*=48	Medical *N*=260	*P* value	Total *N*=326
Pain medicine - No	8(44.4)	20(41.7)	50(19.2)	<0.001	78(24.1)
Geriatric medicine - No	5(27.8)	20(41.7)	97(37.3)	0.581	122(37.4)
Rehabilitation medicine- No	1(5.6)	16(33.3)	79(30.4)	0.067	96(29.4)
Active care of the dying- Yes	13(72.2)	34(70.8)	216(83.1)	0.092	263(80.7)

Figures in parenthesis are in percentage

Two thirds of students felt that morphine improves quality of life, of which the nursing students differed in opinion and one third of students felt that morphine causes death in all dying patients. Majority of students (76.4%) felt that morphine is the universal panacea for all kinds of pain and only less than one thirds of students knew about the role of morphine in relieving breathlessness of heart failure [Table 5].

**Table 5:** Morphine

Question – Appropriate response	Nursing *N*=18	Allied health *N*=48	Medical *N*=260	*P* value	Total *N*=326
Causes death in all dying patients - No	12(66.7)	29(60.4)	182(70)	0.417	223(67.8)
Improves quality of life -Yes	5(27.8)	29(60.4)	181(69.6)	0.001	215(66)
Relieves all kinds of pain - No	2(11.1)	13(27.1)	62(23.8)	0.389	77(23.6)
Relieves breathlessness in heart failure -Yes	6(33.3)	22(45.8)	73(28.1)	0.049	101(31)

Figures in parenthesis are in percentage

Among students participated, three fourth of them acknowledge constipation and drowsiness, and half of them acknowledge nausea and vomiting as common morphine side effects. Around two thirds of the students think that morphine addiction is common in a palliative care setting [Table 6].

**Table 6:** Common side effects of morphine in palliative care setting

Question – Appropriate response	Nursing *N*=18	Allied health *N*=48	Medical *N*=260	*P* value	Total *N*=326
Nausea and vomiting -Yes	13(72.2)	31(64.6)	109(41.9)	0.001	153(46.9)
Constipation - Yes	8(44.4)	24(50)	210(80.8)	<0.001	242(74.2)
Drowsiness - Yes	11(61.1)	36(75)	204(77.3)	0.292	251(76.99)
Addiction - No	2(11.1)	10(20.8)	24(9.2)	0.062	36(11)

Figures in parenthesis are in percentage

Around two third of students could appropriately identify non pain symptoms in palliative care settings [Table 7].

**Table 7:** Common non-pain symptoms encountered in palliative care

Question – Appropriate response	Nursing *N*=18	Allied health *N*=48	Medical *N*=260	*P* value	Total *N*=326
Delirium - Yes	16(88.9)	29(60.4)	184(70.8)	0.073	229(70.2)
Constipation- Yes	6(33-3)	20(41.7)	177(68.1)	<0.001	203(62.3)
Vomiting-Yes	11(61.1)	20(41.7)	193(74.2)	<0.001	224(68.7)
Breathlessness-Yes	15(83.3)	30(62.5)	166(63.8)	0.232	211(64.7)

Figures in parenthesis are in percentage

Majority of students have correctly identified the components that constitute good death [Table 8].

**Table 8:** Components of good death

Question – Appropriate response	Nursing *N*=18	Allied health *N*=48	Medical *N*=260	*P* value	Total *N*=326
Pain and symptom management - Yes	16(88.9)	36(75.0)	238(91.5)	0.004	290(89)
Clear decision making-Yes	17(94.4)	30(62.5)	213(81.9)	0.002	260(79.8)
Preparation for death-Yes	14(77.8)	29(60.4)	210(80.8)	0.008	253(77.6)

Figures in parenthesis are in percentage

Greater part of students felt that prognosis, wishes and choices should be clearly communicated and not communicating prognosis could lead to lack of trust. However, around two third of students felt that prognosis should be only informed to family [Table 9].

**Table 9:** Communicating prognosis

Question – Appropriate response	Nursing *N*=18	Allied health *N*=48	Medical *N*=260	*P* value	Total *N*=326
Prognosis should always be clearly communicated - Yes	15(83.3)	37(77.1)	221(85.0)	0.393	273(83.7)
Prognosis should only be informed to family members -No	10(55.6)	22(45.8)	96(36.9)	0.176	128(39.3)
Not communicating prognosis could lead to lack of trust - Yes	14(77.8)	35(72.9)	218(83.8)	0.175	267(81.9)
Patient’s wishes and choices should be clearly communicated -Yes	18(100)	37(77.1)	212(81.5)	0.093	267(81.9)

Figures in parenthesis are in percentage

Medical social worker, nurse, occupation therapist were correctly identified as part of palliative care multidisciplinary team by majority of students along with radiotherapist who is seldom part of a multidisciplinary palliative care team [Table 10].

**Table 10:** Palliative care multidisciplinary team comprises of

Question – Appropriate response	Nursing *N*=18	Allied health *N*=48	Medical *N*=260	*P* value	Total *N*=326
Medical social worker -Yes	18(100)	36(75)	227(87.3)	0.016	281(86.2)
Nurse -Yes	18(100)	35(72.9)	234(90)	0.001	287(88)
Radiotherapist - No	4(22.2)	6(12.5)	27(10.4)	0.298	37(11.3)
Occupational therapist - Yes	14(77.8)	34(70.8)	192(73.8)	0.836	240(73.6)

Figures in parenthesis are in percentage

## DISCUSSION

Understanding the existing level of palliative care knowledge and attitudes toward end of life care would be an important benchmark for analysis of future educational effort.[2] Medical students and other health care students across the globe are still uncomfortable facing death and dying. The prevailing medical culture continues to view death as a medical failure. Palliative care, despite its growing scientific base, is often perceived as not important. Many trainees do not view palliative care skills as core clinical competencies. These attitudes coalesce into practice patterns that tend to devalue the provision of palliative care even though there is an increasing need for humane medical care at the end of life.[3]

The current health care curricula have inadequate inclusion of palliative care content, skills, and service delivery models in courses, teaching and minimal inclusion of end-of-life content in healthcare textbooks. The attitudes of health care teachers also contribute to deficient palliative care knowledge as dying patients are viewed as not good “teaching cases” and undergraduate students have seldom exposure to a dying patient.[4]

Patients and families bear the burden of suffering from inadequate end-of-life care. The public’s trust in medicine also suffers from concerns that for patients who are approaching death, pain remains unrelieved, and the care provided falls short of patient and family expectations. Interns, residents and other health care professionals need to be able to deal with end-of-life care in a professional and sensitive way that meets the needs of the medical team, family, and most importantly the patient.[5] The SUPPORT study, a study of care of seriously ill hospitalized patients in five teaching hospitals in the United States, has confirmed substantial shortcomings in communication and decision-making in end-of-life settings.[6] A survey of medical students, residents, fellows, and attending physicians found that, there was lack of standardized training in dealing with terminally ill people.[7] In the 1999 Medical School Graduation Questionnaire, 28.2% of students felt that curricular time devoted to death and dying topics was inadequate.[8] A study involving fourth-year medical students regarding preparedness to discuss end-of-life issues with patients found that a majority of students felt unprepared for such discussions.[9] An analysis of palliative care education in the undergraduate medical curriculum found considerable evidence that current training is inadequate in dealing end of life issues, most strikingly in the clinical years.[10] Medical textbooks fall short of providing relevant information for clinical management of terminal illnesses.[11] End-of-life and palliative care education in medical school curricula stand at a crossroads. Despite consensus that these topics merit systematic instruction throughout medical school training, undergraduate medical education lacks standardized curricula for learning palliative care and humane care for the dying.[12]

In this study, the above-mentioned results clearly depict gaps in palliative care knowledge among undergraduate health care students. Appropriateness, outcomes, role of family involvement and legal aspects of resuscitation in a palliative care setting are poorly understood. Significant number of students felt that hastening death or prolonging life is what palliative care believes in. Most of the students think that all dying patients need palliative care and its role in managing non pain symptoms in non cancer setting is inadequately realized. Palliative care is often synonymously compared and considered as pain medicine, geriatric medicine and rehabilitative medicine. Students do feel that morphine causes death in all dying patients and its role in improving quality of life and its role in nonmalignant conditions such as end-stage heart failure is poorly recognized. There is inadequate understanding of morphine side effects and majority of students participated in this study believed that administering morphine in a palliative care setting could lead to addiction. Knowledge of non-pain symptoms in a palliative care setting was not well identified. Students had a reasonable good knowledge in identifying components of good death and communicating prognosis but bulk of students felt that the prognosis should be only communicated to the members of family. The findings of this study clearly reflect the current knowledge and understanding of palliative care among undergraduate students. Quite simply, students are unprepared and lack expertise in death and dying and end-of-life care because our health care training programs, including those that train doctors who care for many dying patients, fail to hold them accountable for competency in these areas.

Reforming existing health care curriculum to incorporate palliative care education is often faced by many challenges on a number of levels. Curricular reform is often greeted by resistance, as there is inadequate knowledge of palliative care among the faculty who decide the content and structure of health care curriculum. Thus, a process of needs assessment, identifying gaps in knowledge, consultations with the curricula reformers and strategic planning is thought to be an effective catalysts for curricular change.[13] There is a strong need to incorporate palliative care education into primary health care education such that there is an overlap between primary health care and palliative care. The attitudes and competencies required to provide high-quality palliative care overlap substantially with those required to provide excellent primary care. Communication skills, understanding of the patient’s wishes and choices, commitment to comprehensive, integrated care of the patient and family, attention to psychosocial and spiritual concerns, emphasis on quality of life and maximizing function, respect for the patient’s values and goals are all quintessential for providing good primary health care and palliative care.[14] A study has shown that 20 h of classroom and practicum training in palliative care allows medical students to meet specified behavioral objectives, and imparts to the students the perception of adequate training in areas of pain management, palliative, and end-of-life care at the time of graduation from medical school.[15] At present, health care education is under the assumption that teaching students how to treat and care for the diseases would provide adequate clinical training and also will prepare them to care for those who would die from these diseases. Yet, deeper reflection and observations, including the SUPPORT study and others, have demonstrated that this assumption is erroneous, that good end-of-life care is not the standard, but the need of the hour. We hope that this study will open up new vistas of possibility, presence and creativity for integration of palliative care education into health care education such that we realize the vision to ease pain and suffering and nurture growth through positive health-illness experience.

## CONCLUSIONS

1. This study demonstrates widespread deficiencies in understanding resuscitation in palliative care setting, understanding palliative care and its philosophy, pain and nonpain symptom assessment and management, communication, and interdisciplinary care for patients and their families.
2. There is a need to integrate palliative care into health care training and the national medical, nursing and allied health council should structure and implement palliative care education into basic health care training.
3. Focused training in palliative care and end-of-life teaching methods and institutional change strategies can facilitate the reform.
